# Conclusion of Miasm and Contagion

**Published:** 1835

**Authors:** John L. Riddell

**Affiliations:** Adjunct Professor of Chemistry, and Lecturer on Botany in the Cincinnati Medical College


### Art. IV.—Memoir on the nature of Miasm and Contagion.

*Read to the Cincinnati Medical Society, Feb. 3d,* 1836. By John L. Riddell, Adjunct Professor of Chemistry, and Lecturer on Botany in the Cincinnati Medical College.

(Concluded from page 412.)

To use his own language, "the results obtained prove very clearly that, in marshy places, during the precipitation of dew, there is an organic matter deposited with it."

Since most of this memoir was in type, I have made some experiments with a view of detecting the aerial miasm of small pox. A perfectly clean ounce phial was half filled with distilled water; a small glass tube with a capillary orifice was made to terminate near the bottom, the upper and much larger portion of the tube bending horizontally to receive the silver nozle of a delicate pair of bellows. Several turns of gauze were passed around the mouth of the phial embracing the tube, and the whole was securely fixed in an appropriate wooden frame-work.

On the fifteenth of February the apparatus was carried to the city pest house, and under the superintendence of Dr. O. M. Herron, it was placed on a table two or three feet from a small pox patient, just in that stage of the disease when the circumambient air is supposed to be most contagious. The bellows were blown by the nurses pretty constantly, for twelve hours, thus presenting a great amount of noxious air to the distilled water. The apparatus was left undisturbed until it came into my hands three days after, when I made the following experiments:

1. One fourth of a drachm of the water contained in the phial, evaporated very slowly in a watch glass, over an alcohol lamp, left concentric circles of a whitish substance. Upon bringing this residue under the object glass of a good microscope, I discovered that it consisted mostly of long crystals, which shot from each other at right angles. The outer margin of each concentric band was less distinctly crystalline, and evidently contained some other substance.

2. A minute drop of sulphuric acid, (carefully distilled and collected on a glass rod, so as not to leave the slightest trace upon being evaporated from clean glass,) was placed upon some of the residue, (experiment No. 1.) Upon the application of heat the acid became black, and upon complete evaporation a dark stain was left; thus showing the presence of organic matter.

3. Upon adding a drop of pure sulphuric acid to near an eighth of a drachm of the water, and expelling the water by a careful heat, the acid became black. This experiment, as well as the one which follows, was performed upon a piece of Florence flask, rinsed in clean water and then heated to redness over an alcohol lamp, in order to remove every trace of organic matter.

4. A drop of the water hastily evaporated, left a whitish residue, not crystalline to appearance, but consisting of extremely minute grains. Upon the application of a high heat short of redness, it became dark colored, indicating the presence of organic matter, by the charcoal liberated. A still higher heat, in contact with air, removed the dark color and left a mere trace of white adherent powder.

These results compel us to believe, that organic matter was communicated to the distilled water by the air which was transmitted through it. This matter did not exist in the air by virtue of its volatility, else in the first experiment it would have been dissipated by evaporation. It was most likely in the form of organized corpuscles, sustained in the air by their exceeding small size.

I was somewhat puzzled with the appearance of crystals in the first experiment, not expecting to see anything more than a gelatinous substance. Upon examining distilled water subsequently, through which the human breath had been passed for several hours, a tissue of delicate arborescent crystals was left after evaporation. I have only time to offer a crude and premature conjecture. May not these saline matters have been borne mechanically into the air, in the one case, muriate of soda from the perspiration of the patient, in the other, a saline substance exhaled in respiration?

About two drachms of the water through which variolous air had been passed, was hermetically sealed in a clean glass tube, leaving a space filled with air above the liquid, equal to one fifth its capacity. The tube has been kept upright in a situation where the temperature is pretty constantly 72 deg. Fahr. An ash-colored sediment soon appeared, which continues to increase. Examined with the microscope a week after it was first adjusted, the sediment appears to consist of numerous, irregular, partly filamentous, ragged masses, with here and there a perfect globule about twice the size of the globules of human blood. The filaments, which may be seen with the naked eye, are seldom arranged in a regular branching manner. After all, we cannot consider these experiments as decisive; for it is evident that in transmitting air even through a capillary tube, mites and filaments of organic dust from the bed clothes etc., may have been sent into the water and retained.

A great law seems to pervade and control all nature, as we rise above the lifeless aggregate, occult, indeed, and incomprehensible in its cause, but not the less obvious in its effects. I refer to the degeneration of races. One race or variety will flourish and endure for a time; but at length it begins to degenerate, and is finally succeeded by other kindred varieties, which have their day, and in like manner yield their places. From the influence of this grand custom, even man himself is not exempt. The Indian is fast disappearing from the forests and plains of America, and where stood his rude wigwam, or where he made war upon the beasts of the wilderness, the white man builds his house, or taxes the fertile soil for the means of wealth and comfort. The mastodons, plesiosauri, and gigantic cycadeæ, of very ancient times are now unknown upon the earth. They are extinct, and beings of modified structure and different habits have succeeded them. Illustrations of this law are constantly presented for our contemplation, in the limited duration and decline of esteemed varieties of fruits and esculent roots; as in the apple, pear, potatoe and yam.

The temporary predominance of certain insect races, of which there are many striking instances on record, seems to have a relevancy to the present subject. I will merely cite a few facts which came under my personal observation. In the spring of 1833, the leaves of the buckeye (*Æsculus ohioensis*) were infested and devoured to an incredible extent, in Franklin county, Ohio, by the larvæ of a small yellow miller. So far as I know, the buckeye has not been infested since.

Some twelve or fifteen years ago, I well remember, that for a mile or two on each side of the Chenango river, New York, every individual sugar maple (*Acer saccharinum*) was destroyed by the depredation of a large caterpillar, which, neither before nor since, has ever made its appearance in sufficient numbers to attract common observation. No thinking mind, I imagine, will fail to trace a close analogy, between the temporary prevalence and anomalous succession of epidemic diseases, and the occasional appearance, in such vast numbers of these destructive races of insects.

Bearing in mind the modifications of which animal races are susceptible, under the influence of altered circumstances, and recollecting the brief hours and minutes which are sufficient to give full maturity to certain animalcules, we may, perhaps, understand why, when the same epidemics prevail at intervals of many years, they are apt to assume different aspects; being at one time quite mild, at another attended with great mortality; giving origin to symptoms in some instances which are unknown in others.

If, then, we admit the presence of living corpuscles in miasmatic air, there are many who would be inclined to suppose, that these corpuscles must possess a very complex organization, to enable them to maintain themselves in that medium. The presence of external organs equivalent to wings, might, by some, be deemed necessary to effect that end. We have, however, the best of all possible reasons, short of demonstration to the senses, for believing that no such necessity exists:—a reason drawn from mechanical philosophy.

Bodies when of very small dimensions, no matter how dense their texture, encounter a degree of resistance in falling through material media, which essentially retards their velocity. Larger bodies do not experience this resistance in so great a degree, because they present less surface in proportion to their weight. Very minute bodies, in falling through the atmosphere, soon acquire nearly a maximum velocity, which they do not exceed until they reach the earth. Dr. Thompson, in his late work on heat and electricity, says that globules of water, the thousandth part of an inch in diameter, acquire by falling through the air, a maximum velocity of nine or ten feet per second. Recognising the laws which regulate the descent of bodies in resisting media, and presuming the miasmatic corpuscles to have the specific gravity of water, I have made calculations to determine the velocity with which one of the monads, measured by Errhenberg, would be capable of falling, through such a medium as common air. The diameter of these creatures is

$\frac{1}{24000}$ of an inch:—one of them would fall near four feet per second.* A corpuscle $\frac{1}{1440000000}$ of an inch in diameter, would fall one inch per second, or five feet in a minute's time. It is therefore easy to imagine, that wingless beings, of transcendent minuteness, may float securely in the subtle air, or be borne on the wings of the winds to the most remote regions of the earth.

In the winter of 1833, I prepared several bottles of water, from clean, recently fallen snow. They were tightly corked, and kept for three or four months in the shaded corner of a room, where the water was not liable to be frozen. At the expiration of this time, having occasion to use some of the water, I observed that the lower portion of each bottle was traversed by myriads of delicate, dark colored filaments, bearing a close resemblance to some of the fresh water algæ. Upon removing the cork, a most unpleasant odor was exhaled, similar to that of animal putrefaction. No one, I presume will doubt, that the living germs of this curious organization came down from the high regions of the atmosphere, in conjunction with the snow.

The doctrine I have espoused might be elucidated still farther, if need be, by analogies from the vegetable kingdom. Perhaps nearly all the true diseases to which plants are liable, arise from encroachment by parasitic fungi and lichens. The rust which infests the culms of wheat, was found by Sir James E. Smith, to consist of highly organized microscopic fungi. We hardly know a single species of the more perfect plants, on whose mature leaves, may not at times, by careful

* In obtaining these results, gravity is regarded as a constant force, and the air as of uniform density. Put $d = \frac{1}{1000}$ of an inch, $v =$ its maximum velocity, 10 feet per second, $m =$ diameter of the monad, $x =$ maximum velocity of the monad per second.

*Required*, the value of $x$ in terms of $d$, $v$, and $m$.

The falling body acquires its maximum velocity, when resistance = gravity. Resistance is as the square of the diameter and square of the velocity:—gravity is proportioned to the cube of the diameter.

Now, as the elements of resistance $d^2\ v^2$ : to the element of gravity $d^3 :: m^2\ x^2 : m^3$. Therefore, $d^2\ v^2\ m^3 = m^2\ x^2\ d^3$. $x^2 = m\ v^2 \div d$. $x = (m\ n^2 \div d)^{\frac{1}{2}} = 4\frac{1}{5}$ feet, when $m = \frac{1}{24000}$ inch.

examination, be discovered some minute and obscure species of this order; and I question much whether a tree can be found in the forest, on whose bark cannot be seen the spreading and parti-colored lichen.

In like manner do parasitic growths affect animal bodies. Do not warts, cancers, sarcomatous tumors, hydatids, and intestinal worms, possess an animal vitality insulated from that of the individual in whose body they occur? For myself, I cannot but regard them as holding about the same relation to the animal system, as the parasitic fungi and lichens do to the more completely organized vegetables.

The majority of medical writers now living, have expressed their belief in the existence of terrene and paludal emanations, which they suppose to be of a gaseous and inorganic nature. No doubt it will be often repeated, that it is unphilosophical to recognise vital corpuscles, as morbific agents, before they have been demonstrated to the sight. "Show us your corpuscles or animalcules, before you call on us to believe in their existence." In reply, it may be said, that we have infinity on either hand; infinite expansion, and infinite minuteness. The range of man's vision, though aided by all the resources of art, is but a point on an infinite line. As well might the skeptic assert, that there were no worlds, no stars, no globes of matter, save what his feeble vision descried; as that the mysterious attributes of life could not attach to beings invisibly small.

